# Analytical exploratory tool for healthcare professionals to monitor cancer patients’ progress

**DOI:** 10.3389/fonc.2022.1043411

**Published:** 2023-01-09

**Authors:** Zoe Valero-Ramon, Carlos Fernandez-Llatas, Gonzalo Collantes, Bernardo Valdivieso, Antonis Billis, Panagiotis Bamidis, Vicente Traver

**Affiliations:** ^1^ Institute of Information and Communication Technologies - Technological Innovation for Health and Well-being (ITACA-SABIEN), Universitat Politècnica de València, Valencia, Spain; ^2^ Department of Clinical Science, Intervention and Technology (CLINTEC), Karolinska Institutet, Stockholm, Sweden; ^3^ Medical Research Institute, Hospital La Fe, Valencia, Spain; ^4^ Lab of Medical Physics and Digital Innovation, School of Medicine, Aristotle University of Thessaloniki, Thessaloniki, Greece

**Keywords:** process mining for healthcare, cancer, decision support, patients’ progress, interactive, analytical, exploratory tools

## Abstract

**Introduction:**

Cancer is a primary public concern in the European continent. Due to the large case numbers and survival rates, a significant population is living with cancer needs. Consequently, health professionals must deal with complex treatment decision-making processes. In this context, a large quantity of data is collected during cancer care delivery. Once collected, these data are complex for health professionals to access to support clinical decision-making and performance review. There is a need for innovative tools that make clinical data more accessible to support cancer health professionals in these activities.

**Methods:**

Following a co-creation, an interactive approach thanks to the Interactive Process Mining paradigm, and data from a tertiary hospital, we developed an exploratory tool to present cancer patients' progress over time.

**Results:**

This work aims to collect and report the process of developing an exploratory analytical Interactive Process Mining tool with clinical relevance for healthcare professionals for monitoring cancer patients' care processes in the context of the LifeChamps project together with a graphical and navigable Process Indicator in the context of prostate cancer patients.

**Discussion:**

The tool presented includes Process Mining techniques to infer actual processes and present understandable results visually and navigable, looking for different types of patients, trajectories, and behaviors.

## 1 Introduction

Cancer is a primary public concern worldwide and specifically in the European continent, with almost one-quarter of all global cancer cases. However, it is only one-tenth of the world’s population (1). In particular, as presented by Dyba et al. (2), in the EU-27 region, the 2020 estimations revealed that there are approximately 1.4 million new male cancer cases and 1.2 million new female cancer cases. The cancer prevalence during the past two decades has dramatically increased as people get older, with 70% of cases diagnosed in men and women over 50 (3). Incidence rates are strongly related to age for all cancers combined, with the highest incidence rates being in older people (4). There are now 4.4 million older adult cancer survivors who have survived over 5 years beyond their diagnosis, while 2.8 million have survived over 10 years (5). Due to the large case numbers and survival rates, a significant proportion of the population is living with cancer needs due to long-term treatments and is suffering from treatment side effects. Consequently, healthcare professionals (HCPs) must deal with complex treatment decision-making processes (6).

Specifically, patients diagnosed with cancer go through complicated and established procedures, and the decisions made about the treatments are essential due to the adverse nature of cancer and its evolution. In this context, a large amount of data are collected during the delivery of cancer care to witness the process of care received by the patient. Cancer data associated with care start by identifying people with cancer who have been diagnosed or received cancer care in hospitals, outpatient clinics, or other providers who diagnose or treat cancer patients. The information collected includes several categories, such as patient demographics, cancer characteristics, stage of disease, treatment, or outcomes. For example, electronic health records (EHRs) are widely used to store longitudinal data; record vital signs, medications, laboratory values, diagnostic reports, mental states, patient transfers, and other health status parameters; and include all cancer data associated with care. However, once collected, these data are complex for HCPs to access to support clinical decision-making and performance review in general, particularly in the cancer context. EHRs and associated software often present data with static views and texts, which do not reveal cancer’s underlying process, evolution, trend, and behavior in the patient’s disease progression or the similarities among patients’ movements. Moreover, health experts usually have to use multiple tools to gather patient status for a complete health assessment. Therefore, there is a need for innovative tools that make clinical data more accessible to support HCPs in these activities. Accordingly, it is necessary to reinforce HCPs and, particularly, cancer care experts, in comprehending what is happening in a cancer care process or environment, thus translating raw clinical data into knowledge. However, this is not a worthless question.

In this regard, Process Mining is a family of process analysis methods and techniques that addresses the discovery and monitoring (and enhances the understanding) of actual processes by extracting knowledge from data stored as event logs recorded by an information system (7). In healthcare, health information systems record data about the execution of processes in a healthcare organization. The data associated with a process execution can be leveraged to create an event log (8). Process Mining techniques can generate valuable process-related insights to diagnose, treat, and analyze disease progression to improve patients’ health status in the cancer context.

In contrast, data-driven medicine relies on visual information about patients’ trajectories or disease risk pathways. Visual tools that can track patient progress are crucial for clinical data visualization. In this regard, clinical dashboards can visually capture the cross-sectional view of various quality metrics. These metrics can include patient statuses and progress, healthcare delivery measures, and performance improvements for care providers. Thus, clinical dashboards can aid in understanding the critical features of the overall patient process and improve outcomes.

One approach for providing HCPs with access to clinical data is to create the infrastructure and interface for a clinical dashboard to make data accessible in a timely and relevant manner. Clinical dashboards are designed to display data to clinicians that impact the quality of care (9). Moreover, a clinical dashboard that presents process-related insights using Process Mining techniques will cover a double objective, allowing the visual exploration not only of clinical data but also the process-related insight, such as patient progress and evolution. Currently, there is very little literature on clinical dashboards for data feedback to health professionals specializing in cancer care delivery and patients’ progress.

In this context, The LifeChamps project1 aims to harness techniques for Big Data modeling, analysis, and aggregation under a novel context-aware, data-intensive, and large-scale analytic framework toward delivering multi-dimensional Quality of Life solutions for different cancer life champions. The project addresses the main conditions of fragility in post-cancer treatment for older adults. The LifeChamps system comprises three main components, the LifeChamps Dashboard, the LifeChamps Platform, and the mobile application for Quality of Life assessment for cancer patients. This work aims to collect and report the process of designing and implementing an exploratory analytical Interactive Process Mining tool, part of the LifeChamps Dashboard for monitoring cancer patients’ progress using Process Mining techniques to extract knowledge from data and present the execution of actual cancer patients’ processes. The organization of the remainder of this article is as follows. Some state of the art about clinical dashboards and clinical decision support systems is included in *Section 2*. *Section 3* presents the background, including Process Mining techniques and the Interactive paradigm. *Section 4* describes a brief description of the methods and tools employed. The experimental results are presented in *Section 5*. Finally, discussion and conclusions are drawn in *Section 6*.

## 2 State of the art

A clinical dashboard is a tool designed and developed to provide clinicians with relevant and timely information they need to inform daily decisions that improve the quality of patient care. It enables easy access to multiple sources of data being captured locally, in a visual, concise, and usable format (10). Evidence shows that dashboards are associated with improved care processes when end-user input is incorporated and information is concurrent, pertinent, and intuitive. Moreover, there is also documentation that implementing clinical dashboards and Clinical Decision Support Systems (CDSS) that provide immediate access to current patient information for clinicians can improve processes and patients’ outcomes (11). Clinical dashboards are often developed by hospitals or health systems, emphasizing statistical analysis, but with poor integration of machine learning or predictive modeling (12). Increasingly, healthcare organizations introduce the clinical dashboard to measure and optimize their internal processes from a management and administrative point of view (9). However, the care itself could be considered as process abstractions, as it is the sequence of a concrete and temporal care event (visits, exams, etc.) witness of the care the patient receives, and substantial to be included for its analysis in a clinical dashboard.

Nowadays, healthcare organizations are increasingly faced with the challenge of providing high-quality service at an affordable cost due to the increased population growth. Effective management and improving such challenging systems’ performance require identifying and optimizing multiple variables. Currently, there are reporting systems in the healthcare sector. Still, their static nature in most cases has resulted in inconsistent, incomparable, time-consuming, and stagnant performance reports that cannot reflect a clear picture of performance and effectively support healthcare managers’ decision-making (13). Dashboards are data-driven clinical decision support tools that analyze data from multiple sources using easy-to-read, color-coded graphical displays. Dashboards can be used not only to promote data-driven decision-making for clinicians but also to improve adherence to evidence-based practice guidelines from a patient-centered perspective. In some studies, as in the revision of Dowding et al. (9), there is evidence that in contexts where dashboards are accessible to clinicians in an easy way, their use is associated with improved care processes and patient outcomes.

One of the problems of dashboards nowadays is the information overload (14). The increased use of electronic documentation in healthcare settings provides a wealth of data, and dashboards will play a pivotal role in converting these raw data into actionable knowledge. This large amount of information means that all data cannot be presented since the user cannot interpret it. In addition, there are different types of users: clinicians, managers, and policymakers. The dashboards must provide each user with the most helpful information in the most appropriate format. Another significant problem of any dashboard in literature nowadays is these dashboards often only include the analysis from the point of view of classical statistics without any investigation from the disease process perspective. They perform statistical analyses to obtain all the information they present. Through these statistical analyses, they also become capable of analyzing, in a certain way, the evolution of patients over time. However, they are limited when it comes to enhancing complex temporal and evolutionary calculations, such as the trajectory of different types of patients, taking into account multiple variables, grouping patients according to these different trajectories, etc.

In this sense, few works are done in clinical dashboards for clinical decision support to monitor cancer patients’ progress. In their work, Janssen et al. (15) proposed two prototype dashboards for use by health professionals delivering breast cancer care to visualize lymphedema patient cohort and individual patient data. The results showed a proven and effective tool for the individual patients, but the dashboard remained a challenge for cohort data, with no progress or evolution information. In another work (12), the authors proposed the design, development, and prototype of clinical dashboards to integrate high-frequency health and wellness data streams using interactive and real-time data visualization and analytic modalities. It uses R packages for data management, normalization, and producing high-quality visualizations over the web using R/Shiny web server architecture. Again, this work lacks information about the patient’s progress over time or evolution. In their work, Adonizio et al. (16) proposed utilizing a design–think paradigm to develop the Lung Cancer Report Card, a near-real-time interactive dashboard identifying actionable care gaps in all patients with lung cancer. Bajaj et al. proposed the creation of an interactive dashboard that provides easy-to-understand key performance indicators (KPIs) for users from all backgrounds and allows intuitive navigation to underlying reports representing finer granularity using tables created in the SQL Server database rendered using the commercial data-visualization tool Tableau (17). It supposes converting massive amounts of data into actionable information but does not incorporate machine learning capability to infer patients’ progress and evolution. All previous works are good examples of clinical dashboards for data aggregation in the cancer field, presenting data visually and appropriately and incorporating statistical analysis. However, they do not infer processes from real-world data in the cancer field. Clinical processes are complex, diverse, and highly dynamic. Providing healthcare organizations and, specifically, HCPs with knowledge to understand how their patients’ care processes is currently being performed is important to detect gaps, inefficiencies, or improve such processes (18). By discovering the patient pathway, it is possible for one to have a consistent understanding of the healthcare provided. Healthcare information systems generate event log data tracking patient-care processes supposing a valuable data source for analyzing and studying the processes (19). Therefore, incorporating such analysis could be a great opportunity in cancer field as treatment pathways and care are also recorded in healthcare information systems. Then, this information is sensitive to be analyzed as a process to comprehend it better.

### 2.1 Co-creation with end-users

When talking about clinical dashboards and tools, it is essential to consider end-users’ expectations, needs, and requirements to ensure their acceptability and use. Following co-design and co-creation methods during the design and development phases might be a good approach (20). Potential end-users (clinicians, health managers, and researchers) should be incorporated into the loop to consolidate their needs and requirements.

However, it is not only a question of incorporating end-users’ needs and requirements when developing a clinical tool. It is even more critical to ensure its appropriateness and understandability of the results. The healthcare domain has its particularities. In particular, it implies a theoretical knowledge about the process under study needed to develop any clinical tool for a concrete context. For that, the health experts’ involvement is paramount. When talking about clinical decision support, health experts should not only comprehend and trust the tool’s decision but also have the possibility of correcting and improving the results based on their awareness. Moreover, they are a decisive source of knowledge and expertise about the cancer care process to be considered and integrated into a clinical dashboard’s results.

The Interactive paradigm defines this concept by integrating human activity into the process (21). It assures a close collaboration between the tool and the human to provide models that professionals can use to understand the actual process better and allow results’ correction and improvement according to human knowledge and experience. In this context, it is explicit that applying the Interactive paradigm in the definition and development of a tool requires the continuous involvement and engagement of human experts in the learning process. It means strengthening the acceptance of the entire methodology and its results by professionals.

## 3 Background

As explained in *Section 4*, the medical experts’ involvement is needed in the overall process of co-designing the tool to incorporate human knowledge as another input in the analysis. The tool should support HCPs in formulating questions with clinical relevance to gain understanding and knowledge about cancer patients’ processes. It is paramount to propose a working framework that allows HCPs to inquire about what they want to analyze from the underlying processes and how they want to visualize them instead of presenting results without their involvement and comprehension.

### 3.1 Interactive process mining paradigm in healthcare

As introduced, the Interactive paradigm defines the concept of incorporating human activity into a process. Using the Interactive paradigm ensures a co-creation process for the tool design and implementation in collaboration with the end-users. In this line, the work presented in Fernandez-Llatas (22) proposes applying Process Mining techniques over the Interactive paradigm to infer healthcare processes adhered to by patients using feasible Process Mining algorithms. This information is appropriately displayed in formal workflows. HCPs could filter and evaluate these workflows by exploring new medical evidence.

#### 3.1.1 Interactive process indicators

Within the Interactive Process Mining methodology, an Indicator is any *information that helps to understand or measure the characteristics or intensity of one fact or even to evaluate its evolution*. Thus, a Process Indicator (PI) is a *process representation that can be used as an indicator to understand or measure the behavior of a process* (22). It means IPIs use the benefits of the Interactive framework to create process-based indicators that provide human-readable and contextualized KPIs using a co-creation approach. ts of the operations, going from the general to the individual. Consequently, IPIs are not numbers but advanced views providing human-understandable information supporting experts using better processes’ perception and assessment of the processes going from the general to the individual. IPIs are not numbers but advanced views in the form of enhanced processes that provide a human understandable view that supports the expert for the better perception of the processes for an advanced assessment.

### 3.2 Process mining tools

Process Mining (23) is a relatively young research discipline that focuses on extracting knowledge from data generated by any process stored in a database. Process Mining provides tools, algorithms, and visualization instruments to allow human experts to obtain information about the characteristics of the execution of a process by analyzing the trace of events and activities that occur in a concrete procedure from a process-oriented perspective. Its application over the Interactive paradigm results in the Interactive Process Mining paradigm in the healthcare domain.

Several examples of Process Mining applications to the healthcare domain include the work presented in Yoo et al. (24) that utilized EHR data for evaluating the hospital processes using a Process Mining technique. Moreover, Mans et al. proposed two studies using Process Mining techniques in healthcare: Mans et al. (25) presented a study for the gynecological oncology process from three different perspectives, the control flow, the organization, and the performance, and Mans et al. (26) conducted a study involving process-related information for stroke patients.

In the current framework, we can find a set of commercial Process Mining techniques and algorithms that can be applied to an event log to generate models, tables, and data for analysis, with different results, capabilities, and characteristics in the healthcare domain. Exploring the event logs associated with EHR and related to cancer treatment using Process Mining is a promising way to support the comprehension and improve the quality of cancer care processes (25, 27, 28).

## 4 Materials and methods

As introduced in previous sections, HCPs’ involvement is crucial when producing a clinical tool. Therefore, a framework for applying the Interactive Process Mining paradigm is needed to bring it to the end-users. Hence, they work with the framework to understand their processes with the final objective of co-creating PIs. This methodology should support HCPs in what they want to analyze, enabling data analysis interactions between process miners and HCPs to transform raw data into comprehensive insights and information. Fernandez-Llatas defined these interactions as *Interactive Process Mining Data-Rodeos* in the Interactive Process Mining paradigm context. A Data-Rodeo is a *highly coupled multidisciplinary interactive data analysis aimed at building Process Indicators that allow understanding, quantifying, and qualifying processes and their changes in an objective, comprehensive and exploratory way* (22).

### 4.1 Interactive data-rodeo methodology: A co-creation process

**Figure 1:**
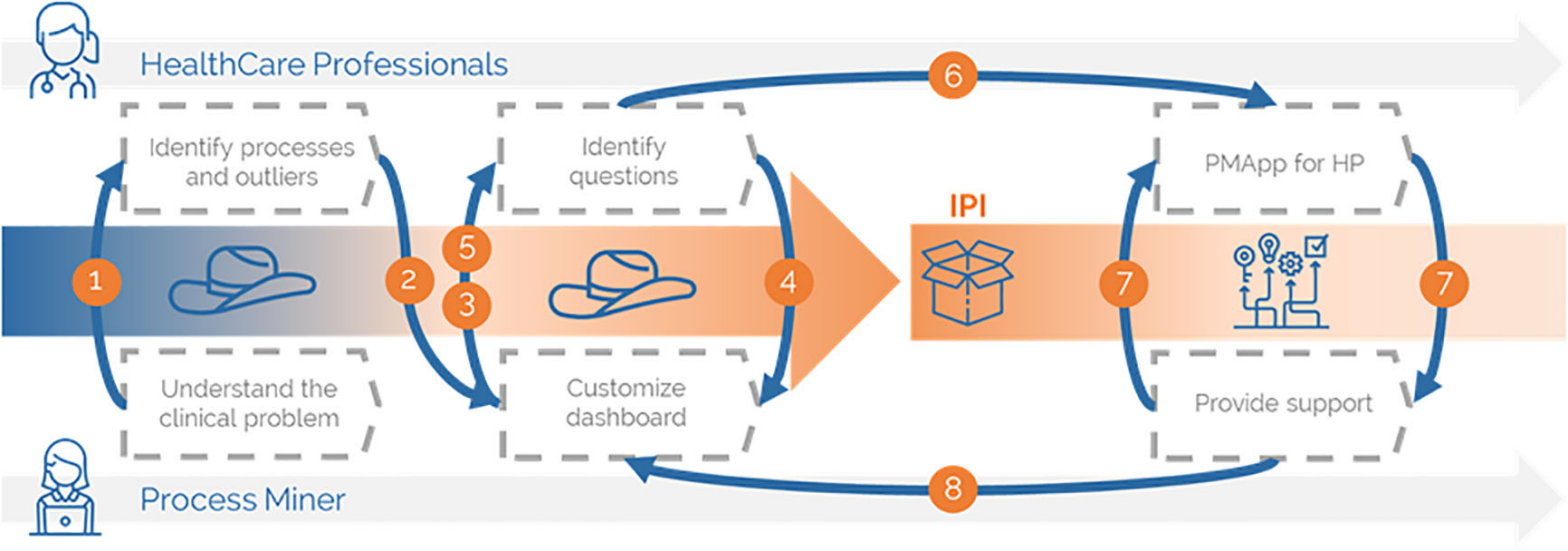
Performing a Data-Rodeo.

From a high-level description, a Data-Rodeo describes how the process miners and HCPs interact until an IPI is wholly built (see **Figure 1**). As a result, not only a set of PIs are developed, but also a customized Process Mining exploratory tool for a concrete clinical question. Overall, process miners deal with the data, including availability, ingestion, and quality, and show first views to the HCPs. Secondly, HCPs identify processes and start analyzing problems with the data and concrete cases that need special care. With this information, the process miner acquires a better understanding of the actual processes and the clinical problem. HCPs identify perceptual questions based on their daily experiences that the process miner translates into advanced beta views, helping them distinguish between more relevant clinical questions. Then, these novel views might support identifying new information, such as outliers, which will become further questions and hypotheses being enriched or generating unique advanced displays. Finally, these breakthrough views are presented to HCPs to be validated. This process may take several iterations until the IPI is entirely built. The process concludes with the IPI and the personalized tool to explore with.

When the user is set at the center, specifically with expert knowledge about the clinical processes but with little to no Process Mining abilities, interactive and visual tools are needed. For our problem, we chose the PMApp tool because of its versatility and proven performance in healthcare scenarios, and because it facilitates the Interactive Process Mining paradigm. PMApp expedites producing interactive and exploratory tools that respond to the selection of arrows and nodes by capturing Graphical User Interface (GUI) events. It also allows the user to create custom forms and algorithms for discovery, custom filters, and enhancement maps (29). It permits the traceability of all learning processes, so each activity is continuously associated with single events. In PMApp, it is also possible to enhance the discovered model using color gradients. With this feature, it is possible to generate specific maps that highlight particular situations that depend on a customized formulation represented by nodes. All previous characteristics show that HCPs comprehend the processes in a better manner. Furthermore, the PMApp framework has been widely tested in real healthcare scenarios, such as in the analysis of the follow-up protocols of patients with diabetes (30, 31), the characterization of emergency flows, or for measuring organizational changes effects (29), among other works. This characteristic and the possibility of creating a custom dashboard made the difference when selecting PMApp as the base tool for developing the dashboard in the present work.

**Figure 2:**
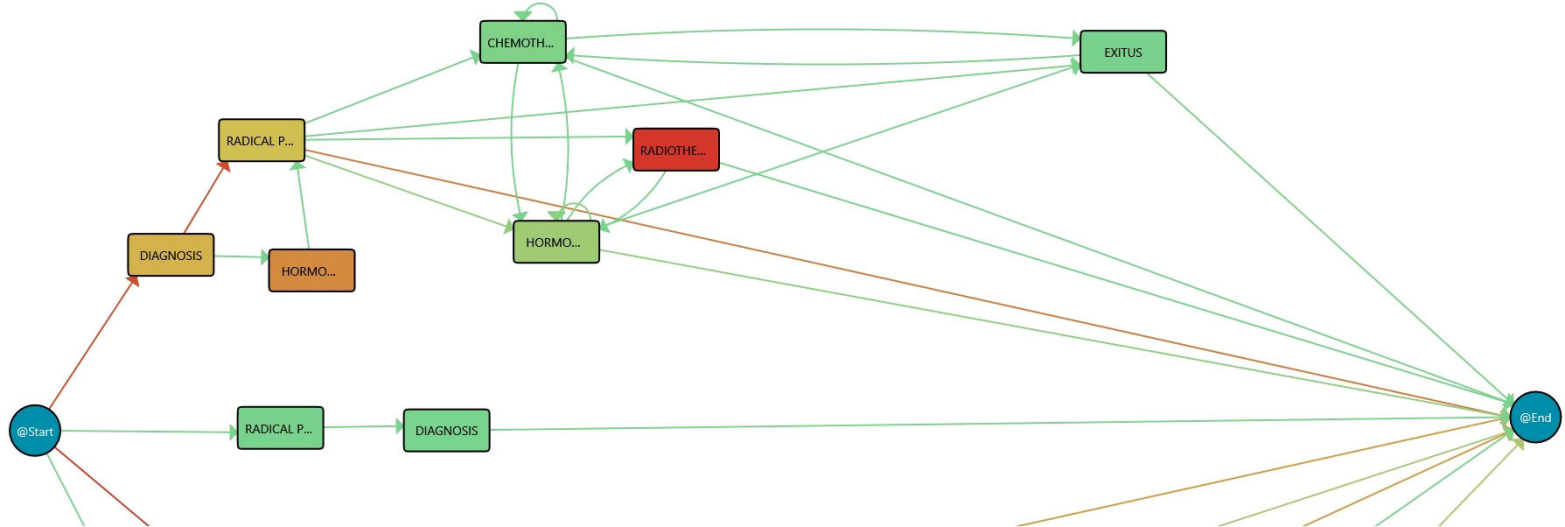
Process Mining rationale. Redder color in nodes, as opposed to green, represents higher time in that stage while redder color in transition means larger number of cases in that transition.

With PMApp, the processes with the data are represented as Timed Parallel Automata (TPA) as workflows representing the different events systematically, their connections, the time spent at each activity, and the flow that was followed; all were summarized in a specific workflow, as seen in the example included in **Figure 2**. This powerful visualization is called the IPI and is explained in the following lines. In **Figure 2**, the events (nodes) that exist, as seen in the IPI, are as follows: (1) “Artificial Start” (@Start) represents the initial event always present; (2) Diagnosis; (3) Radical Prostatectomy; (4) Hormonal Therapy; (5) Chemotherapy; (6) Radiotherapy (all previous representing the different care steps of a prostate cancer patient); (7) Exitus (deceased); and (8) “Artificial” End (@End) is always present as the final event. In the IPI, it is observed that the patient can have different treatments, different destinations, etc. A transition is needed so that a care episode can be traced; e.g., a patient is treated with hormonal therapy after a radical prostatectomy. This is represented by changes (arrows) between the nodes. Essential information resides in the time spent at the nodes and the different paths the visits go through. Summarized information about, e.g., the number of treatments, the time between treatments, etc., can be seen through color gradients in the IPI for the nodes and transitions. In this specific case, there is a green-to-red gradient for the nodes representing the median duration in the node (e.g., time spent at diagnosis) and for the transitions showing the number of patients traversing this concrete step. As said, PMApp offers a set of algorithms, filters, and enhancement maps together with the possibility of creating new ones based on the specific characteristics of the clinical problem and the HCPs’ needs. Thus, PMApp is the point of departure for our work. It should be customized and adapted to the HCPs’ needs participating in the LifeChamps project and the context of monitoring cancer patients. Therefore, the results presented in this work are the customized and exploratory Process Mining tool, adapted to the LifeChamps project particularities (data ingestion, data sources, etc.) and an IPI to monitor prostate cancer patients’ care process.

Following the Data-Rodeo framework, a set of steps and interactions between process miners and HCPs were planned following three phases:

- Preparation phase. It comprises the first contact with the HCPs when the scope of the co-creation process and steps to follow are explained, and data access is provided.
- Research phase. This phase looks for tailoring PMApp through the definition of an IPI during the subsequent Data-Rodeo sessions to support HCPs.
- Production phase. HCPs use the analytical and exploratory tool in their daily practice.

In the LifeChamps context, eligible HCPs were multidisciplinary and involved in cancer care for “older” adult cancer survivors diagnosed with breast cancer, prostate cancer, or melanoma. In addition, we used actual data from a particular project partner, Hospital La Fe in Valencia, a publicly owned and managed hospital responsible for the healthcare of 300,000 inhabitants. The data were collected retrospectively from the EHR from May 2011 to April 2022 from 1,267 unique patients with a prostate cancer diagnosis. We choose to start with patients diagnosed with prostate cancer because of two reasons. On the one hand, it was within the three LifeChamps project use cases (breast cancer, prostate cancer, or melanoma). On the other hand, the hospital urologist was especially committed to applying such techniques to investigate patients’ processes and evolution.

The records included 11 variables, including age at diagnosis, clinical episodes related to cancer care, episode date, or grade. **Table 1** presents the complete data set variables. Moreover, it should be mentioned that ethical approval was obtained for this study from the Ethical Committee of Hospital Universitario La Fe on 31 March 2021 (Registration number: 2019-157-1). Before their transfer, all samples were entirely anonymized by the hospital’s IT department. During the preparation phase, 57 HCPs participated *via* an online survey or telephone/online interviews, part of the complete multinational study to evaluate healthcare needs, preferences, and expectations in supportive cancer care perceived by cancer survivors, family caregivers, and HCPs carried out in the context of LifeChamps project and presented in Marshall-McKenna et al. (32). During these interactions, the project scope was explained, and their needs for monitoring cancer patients’ progress were collected. **Table 2** shows the characteristics of the participants. The study includes a complete description of all stakeholders’ views, perspectives, and expectations. The most useful feedback for implementing of the tool were as follows: (a) HCPs participating in the study highlighted the need to incorporate EHR data with the other project data sources; (b) they expressed their concerns about the need for adequate IT support and training in the tool by the project technical team; (c) they agreed on the need for a user-friendly and accessible solution; and (d) they also supported evaluating patients’ cases over time, individually and at a population level. These results worked as inputs for the design of the overall LC Dashboard and, particularly, for the analytical tool presented in this work.

**Table 1:** Data description: Episodes and cohort.

Column Name	Description	Type	Example
Patient ID	Global unique identifier	Numeric	24972
Date	Episode date	Date	18 May 2021
Episode	Episode name	Alphanumeric	Chemotherapy
Type	Detail of drug for Hormonal T. and chemotherapy episodes	Alphanumeric	Abiraterona
Value	PSA value for PSA episode	Numeric	20.94
Age	Patient age at diagnosis	Numeric	65
PSA Range	PSA range at diagnosis (ng/ml)	Alphanumeric	10–20
Grade	ISUP^1^ grade at diagnosis	Alphanumeric	Grade group 1
Tr1	Initial group treatment	Alphanumeric	OBS (Observation)
TR1 detail	Initial treatment in detail	Alphanumeric	ADT + QT

^1^International Society of Urological Pathology.

**Table 2:** Preparation phase participants’ characteristics.

Variable	Value	*n* (%)
Gender	Female	41 (71.9)
	Male	15 (26.3)
	Not specified	1 (1.8)
HCP Role	General practitioner	14 (24.6)
	Clinical nurse specialist	11 (19.3)
	Clinical oncologist	8 (14)
	Urologist	7 (12.3)
	Physiotherapist	5 (8.8)
	Medical oncologist	4 (7)
	General nurse	3 (5.3)
	Psychologist	2 (3.5)
	Specialist radiographer	1 (1.8)
	Dermatologist	1 (1.8)
	Dietitian	1 (1.8)

During the research phase, we closely collaborated with the prostate cancer experts at Hospital La Fe. In particular, we performed three Data-Rodeo sessions. During these sessions, two experts from each field participated: two process miners and two clinical experts (a urologist and a biomedical engineer). At Hospital La Fe, given a suspicion of prostate cancer due to elevated prostate-specific antigen (PSA) or pathological digital rectal examination, an ultrasound-guided trans-rectal or trans-perineal prostate biopsy is performed, taking into account the patient’s life expectancy and comorbidities. When the diagnosis is confirmed, the patient is classified according to the risk of the disease (considering the Gleason grade, the PSA value, and the tumor extension), and the most appropriate treatment options for the patient are considered. This information is included in the actual data set used (**Table 1**). The main treatment groups are as follows: Active Surveillance, Radical Prostatectomy, Radiation Therapy, Hormone Therapy, Chemotherapy, and Observation. Once the treatment or combination of previous neoadjuvant, concurrent, or adjuvant treatments has been established, the disease’s response and progression are monitored biochemically (monitoring PSA levels) and radiologically (using imaging tests). Applying Process Mining techniques in this context is of great interest to characterize the most effective lines of treatment for each patient profile, determine the most optimal duration and combination of treatments, identify prognostic factors of disease progression, and monitor proper compliance with the monitoring protocol and alerting of loss of monitoring or risk patterns. Thus, the IPI goal was to *comprehend the prostate cancer care process better*. In this regard, the patient process information could help clinicians to study the patient flow behavior and how they are expected to evolve. Accordingly, three sessions were performed:

- First session. On the one hand, the aim was to understand the data by the process miner and the clinical problem behind it. On the other hand, explain the rationale behind the tool to the HCPs.
- Second session. This session was devoted to explaining the first version of the tool and the IPI in a live session and collecting in-depth clinical experts’ feedback, knowledge, and concerns to be incorporated into the following versions of the IPI and the tool.
- Third session. During this last session, the technical team presented the improvements in the tool and IPI.

After each session, the technical team worked with data and produced refined versions of the tool and the IPI. The consecutive sessions had two main goals: the HCPs’ feedback acquisition to refine the tool and the IPI and, secondly, to foster the learning process that will facilitate the tool utilization by the end-users during the production phase. The last phase, the production phase, will be implemented during clinical trials in the context of the LifeChamps project. Thus, the next section of this work presents the results from the first two phases in the form of an analytical exploratory Process Mining tool to monitor cancer patients’ progress and an IPI for the prostate cancer care process.

## 5 Results

Following the co-creation and interactive approach thanks to the Interactive Process Mining paradigm in healthcare defined in *Section 6*, we developed an Analytical Interactive Process Mining tool to monitor cancer patients’ progress to be integrated within the LifeChamps (LC) Dashboard and an IPI for the prostate cancer care process. The IPI is integrated within the tool to allow its analysis by the HCPs. Overall, the LC Dashboard presents to HCPs the evolution of the patients. It is intended to be used by HCPs, clinical researchers, and clinical managers in the field of cancer. The LC Dashboard includes two sets of information based on their approach.

### 5.1 Classic approach

The LC Dashboard incorporates classic analytic tools for patient monitoring as other dashboards, as presented in *Section 4*. It enables HCPs to visualize and monitor relevant and timely information to inform daily decisions. This information includes self-report by the patients, and it includes individualized predictions about frailty and quality-of-life subdomains and ill-health transitions with an emphasis on the identification of fit, pre-frail clinically, and frail older cancer survivors, these models for frailty and quality of life particularized for each patient., sleep monitoring, breathing rate, skin temperature and SpO2), a smart scale (measuring weight and body composition), home sensors (tracking ambulation and functioning).

### 5.2 Analytical interactive process mining tool for monitoring cancer patients’ progress

**Figure 3:**
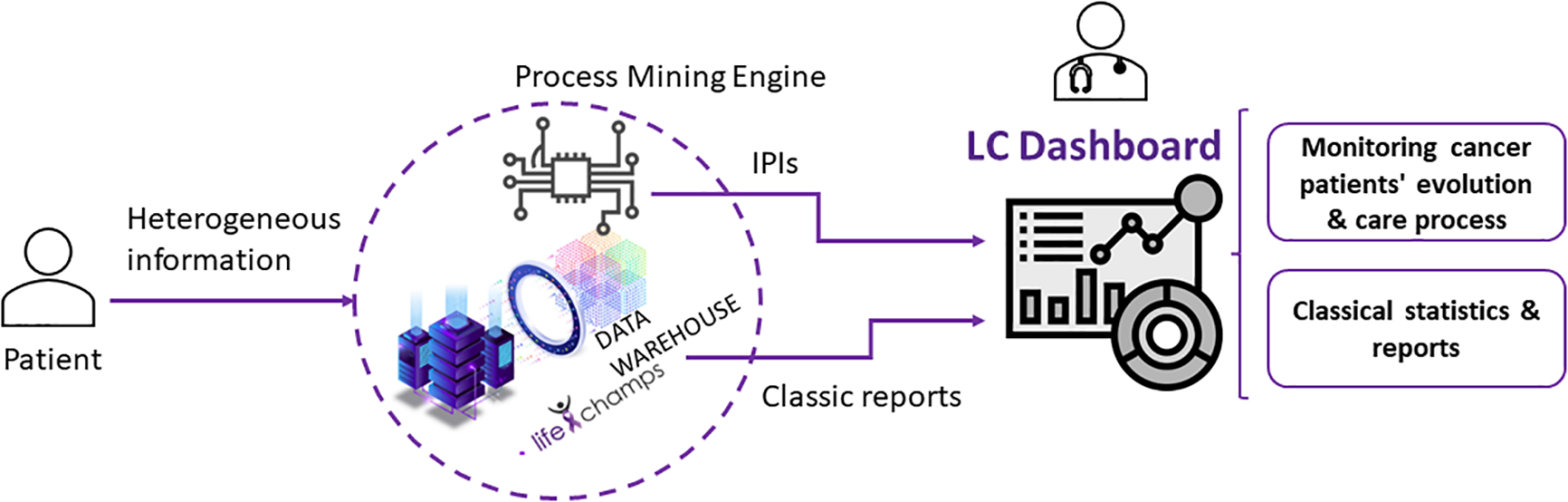
Dashboard logical diagram.

It deals with the interactive analysis of cancer patients’ processes. It includes the results based on the process-oriented data analysis using the Interactive Process Mining methodology. The tool has been developed based on the PMApp framework, totally adapted for the purpose of this study. Specifically, we (a) develop the data ingestion mechanism to be compliant with the LifeChamps infrastructure and data sources, (b) develop the user interface, (c) develop new filters and algorithms needed for the analysis and to communicate with the LifeChamps infrastructure, and (d) adapt needed PMApp components to the project context. **Figure 3** presents the LC Dashboard logical diagram within the LifeChamps project. Using heterogeneous information from the patient—clinical, demographic, sensors, and self-reported information—the Analytical Interactive Process Mining tool might present understandable information with clinical value to evaluate patients’ progress over time and evolution. This information is presented as a set of graphical and navigable IPIs. These IPIs and the Analytical Interactive Process Mining tool for monitoring cancer patients’ evolution are the result of applying the Data-Rodeo methodology with the HCPs involved in cancer patient care and management presented in *Section 6*. *A* summary of the main results from the Data-Rodeo methodology through the three performed sessions is included in **Table 3**. The table presents the objective of each session, a summary of the collected feedback, and how it fed the next iteration of the methodology, the IPI, or the tool. Overall, during the different sessions, experts from the hospital gained an understanding of the tool and the IPI, which was translated into insightful feedback and a refined IPI.

**Table 3:** Summary of main results collected during the Data-Rodeo sessions.

Session 1	
Objective	Understanding the data and the clinical problem A first approach to the tool
Feedback	Data - episodes: most important care episodes since the diagnosis of the cohort study Data - study cohort: information at diagnosis How prostate cancer is treated at the hospital
Application	IPI design Data ingestion: what data to be used What components from PMApp to be incorporated What new components should be developed
Session 2	
Objective	Explain the preliminary IPI and the tool in detail using the IPI as a guiding thread Gain an understanding about the variable PSA and its incorporation to the IPI
Feedback	HCPs were interested on grouping treatment options A better understanding of the disease by the technical team is needed Detection of errors: patients with treatment after exitus, and treatments before diagnosis PSA evolution: presented results considered the PSA as a sole variable, but HCPs wanted to see PSA evolution combined with the treatment information Color palettes for heat/differences maps are difficult to interpret by color blindness
Application	Data curation HCPs provided with more details about the PSA clinical meaning and use New developments for incorporating the PSA evolution after treatment and between treatment episodes Development and implementation of five new color palettes for color blindness users
Session 3	
Objective	Presenting the refined version of the IPI with errors corrected and the PSA evolution combined with the treatment care pathway
Feedback	PSA evolution is useful and meaningful Grouping functionality is very useful to play with
Application	HCPs should play with the tool and the IPI before considering any new change

The following section describes the tool using the implemented IPI for the prostate cancer care process as a guiding thread.

### 5.3 IPI: primary process

The implemented dashboard includes graphical representations of the IPI for its analysis. Very briefly, *Section 5* revealed that Process Mining technologies use a log of actions recorded on a temporal basis to infer workflows that explain the whole process in a human-understandable manner. Process Mining understands data as recorded event logs, where each event refers to a case, an activity, and a point in time to discover, monitor, and improve actual processes. Each *event* contains timestamp information about a patient’s healthcare episode. A set of events corresponding to the same patient is called *case*. Furthermore, a *log* is a set of cases. In this case, the PALIA Discovery algorithm produces the process flow behind the considered log, as shown in **Figure 2**.

Overall, the progressive views of the IPIs are the models that represent the current processes’ status. These models can be enriched to highlight relevant information (enhancement). Moreover, a timely stratification comparison between models can be made to support experts in understanding the applied changes (evolution) and grouping similar behaviors (clustering). Nevertheless, other views can provide a high semantic statement of the findings (abstractions), such as numbers describing an objective measure acquired from the models that graphics can represent. Progressive views can allow experts to navigate models to discover their root causes.

**Figure 4:**
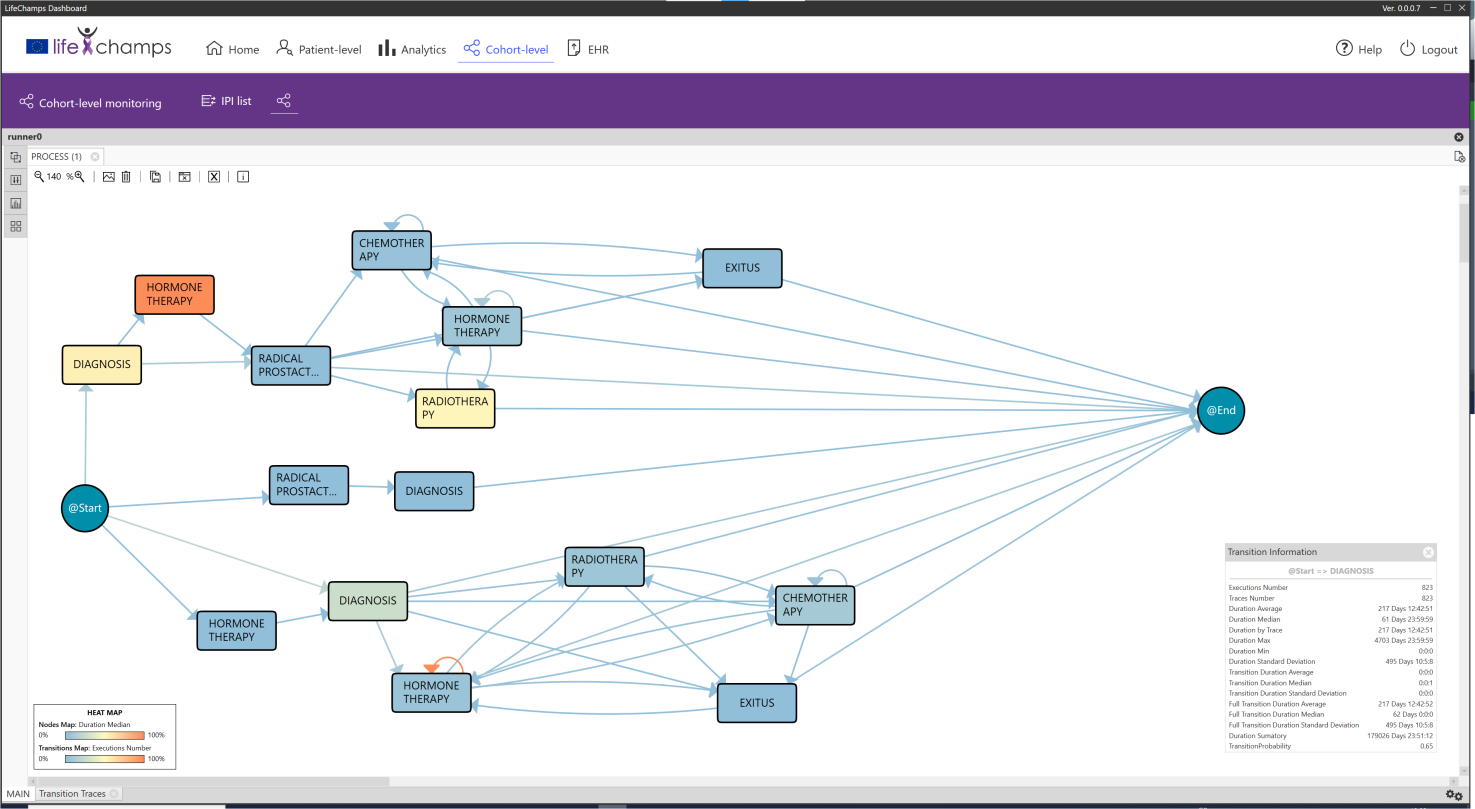
IPI: main process. IPI representing the care episodes of 1,267 patients with prostate cancer diagnoses. More orange color in nodes, as opposed to blue, represents higher time in median in that stage, while more orange color in transition means larger number of cases in that transition as specified in the heat map legend (also included in the figure).

Based on the information provided by the IPI, the analytical exploratory Process Mining tool of the LC Dashboard shows different perspectives about the process with various focuses. In particular, it displays information that allows understanding the population under study, the possibility of augmenting the reality with other tools, and navigating from the process to the individual, as included in the following sections. Following the IPI for the prostate cancer care process, **Figure 4** includes the discovered process for prostate cancer patients’ care. The IPI’s main view shows the care process with the different clinical events (diagnosis, radical prostatectomy, chemotherapy, radiotherapy, hormonal therapy, and exitus) and the transitions among them. We can differentiate among three processes. The process top *circuit* includes patients with radical prostatectomy and other treatments, including radiotherapy, chemotherapy, and hormone therapy. The middle *path* shows patients with only a radical prostatectomy, whereas the bottom process *circuit* represents patients without a radical prostatectomy. It is worth mentioning that the diagnosis and the first treatment were stored with the same date in the data set. For this reason, on some occasions, the episode event appears before the diagnosis event in the flow. This fact offered insight into how the information is recorded in the clinical consultations and could be refined to improve the process understanding.

### 5.4 Understanding the population: New insights

Apart from the events representing the process or episodes, the cohort data include other information such as socio-demographic facts, laboratory measures, or patients’ self-reported information. This information could be relevant to the process. Therefore, it could be considered for enhancing the IPI with more significant views. In this concrete case, the data set contained the patient’s age at diagnosis, the ISUP (International Society of Urological Pathology) grade at diagnosis, the PSA range at diagnosis, and the initial treatment group (see **Table 1**). The tool analyzes this information to characterize the population under study to present new insights for a better understanding.

#### 5.4.1 Groups

**Figure 5:**
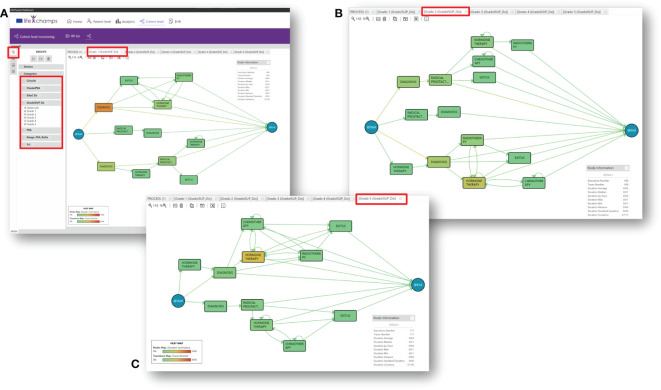
Insights. IPI with groups by Gleason Grade Groups at diagnosis for prostate cancer. **(A)** Gleason Grade Group 1, **(B)** Gleason Grade Group 2, and **(C)** Gleason Grade Group 5.

In particular, the dashboard analyzes this aggregated information from a cohort perspective. Comparing groups is a handy tool that could help HCPs to discover and understand the nature of the differences among groups. During the Data-Rodeo sessions, a set of categories with clinical meaning was identified for its use in grouping the population. These categories were the PSA range and the ISUP group at diagnosis. **Figure 5** includes three of the five discovered groups for the five ISUP grading, Grade Groups 1–5 (33); specifically, **Figure 5A** shows the population diagnosed as Grade Group 1, **Figure 5B** represents the population interpreted as Grade Group 2, and **Figure 5C** shows those diagnosed as Grade Group 5. The tool allows looking for differences, as it can be observed that these three processes are different. For example, Grade Groups 1 and 2 include a population with a radical prostatectomy treatment, whereas this treatment is not present in Grade Groups 3, 4, and 5.

**Figure 6:**
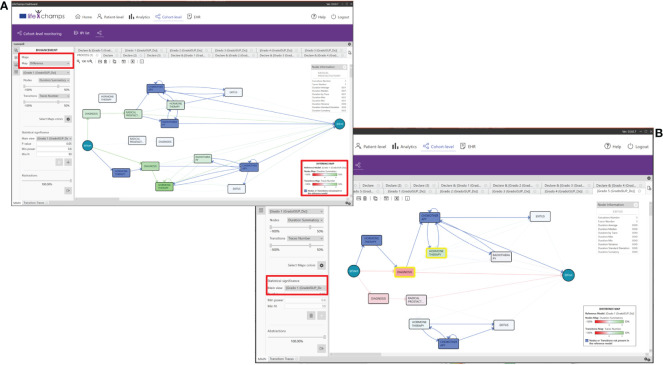
Differences between groups. **(A)** IPI showing the differences between the concrete Gleason Group 1 and all the patients and where the differences lie. Greener color in nodes, as opposed to red, represents higher difference in time spent in median in that stage, while the reddest color in transitions means larger difference in the number of cases in that transition. **(B)** IPI showing the statistical significance between Gleason Grade Groups 1 and 5. Nodes highlighted in yellow represent where the difference is statistically significant.

In this regard, a classic trust measure to evaluate different medical processes is to show differences, known as statistical significance. Overall, it helps to quantify whether a result is likely due to chance or some factor of interest. Thus, it was a perfect candidate to enhance the discovered models and be included in the IPI. Most of the literature focuses on the *p*-value for measuring the statistical significance (34). The dashboard incorporates the statistical significance using *p*-value for comparing nodes that refer to the same process state. Following the literature, the threshold was set to 0.05 (29). This technique can highlight the differences with statistical significance between the two flows of the model. This approach can discover when a process is different and in which parts of the models the differences lie. Following the IPI and focusing on the ISUP groups, we can compare, for example, Grade Group 1 (low-grade cancer) with the rest of the ISUP groups and the general process; the tool analyzes the differences between Grade Group 1 and the others and colors the model following a difference map, featuring the node not present in the model (see **Figure 6A**). Moreover, the tool calculates the statistical significance and adds it to the model by coloring with yellow (see **Figure 6B**). Finally, the *Declare* option allows extracting a group for a concrete event or path. This facilitates the analysis of a definite course within the process. For example, we can analyze the group of patients who had an *exitus* event and look for common characteristics or differences from the rest.

#### 5.4.2 Clustering

**Figure 7:**
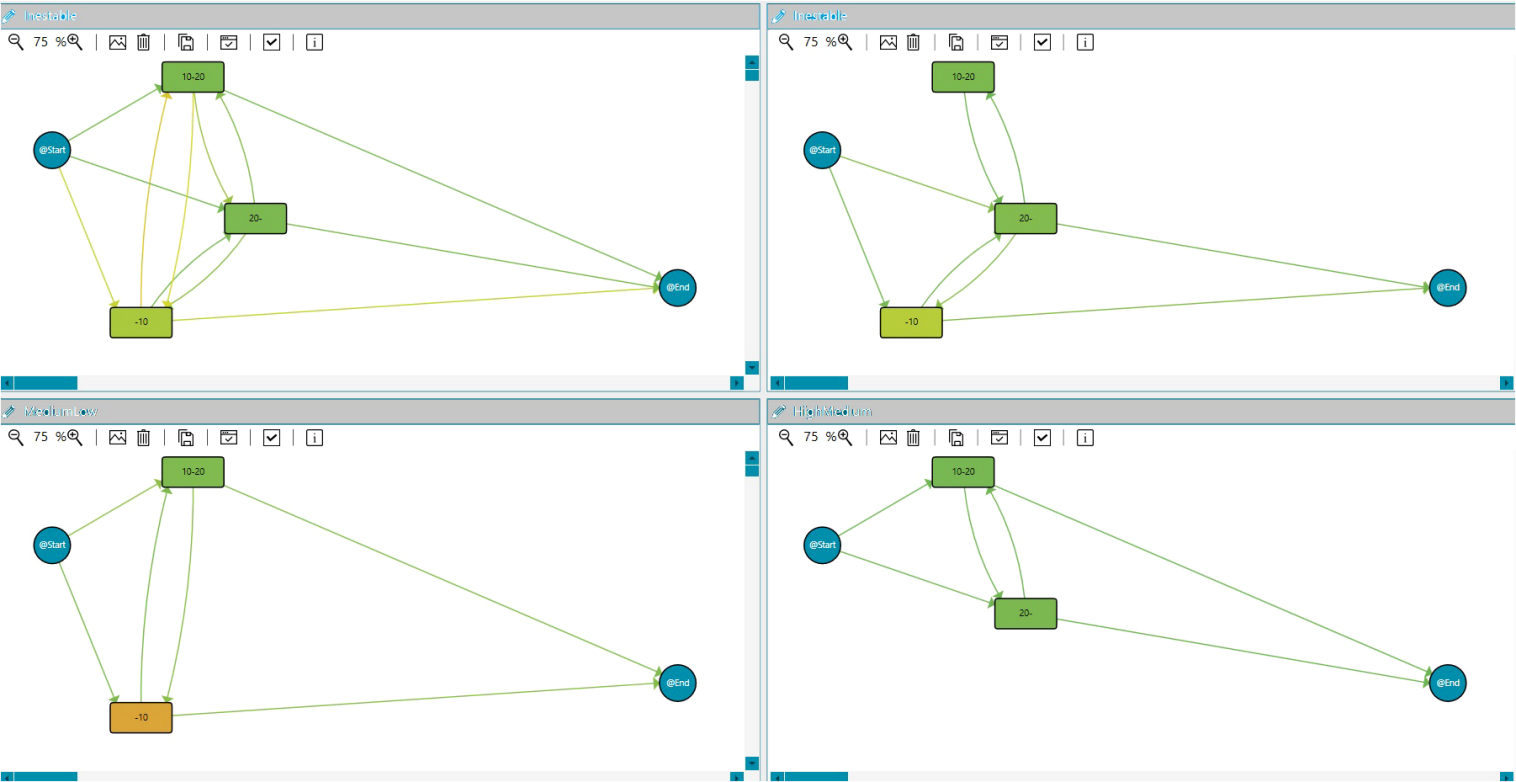
Clustering. IPI showing the clustering models for PSA evolution during the study duration: PSA unstable evolution, PSA low to medium, and PSA medium/high.

Process Mining can construct individual and human behavior models (35). It allows for analyzing health determinants and the variability and evolution of a disease over time. In this framework, trace clustering techniques are unsupervised Data Mining solutions that can group traces with similar behavior, maximizing differences with the rest of the groups. They could be seen as a Process Mining Conformance technique because it uses distances among the models for grouping the traces (36), supporting us in the dynamic approach for stratification groups that permit a better understanding of the clinical cases. In the context of this IPI, extracting information and knowledge from data and discovering patients with different risk behaviors regarding the PSA evolution might be relevant for the HCPs involved in cancer care. Therefore, we incorporated the Quality Threshold Cluster (QTC) algorithm into the dashboard. This algorithm requires a *quality threshold* to determine the maximum distance among traces in the cluster. s for diverse chronic diseases. It builds *k* partitions from the entry sample, although its main disadvantage is that the *k* parameter is fixed, forcing the expert to primarily decide the number of groups. We also implemented three main distances for the clustering algorithm, Topological (30), Heuristic (36), and Levenshtein distances (37). **Figure 7** includes the results for the Quality Threshold Clustering and topological distance for the PSA evolution. The most meaningful results were obtained for a threshold of 0.3 with seven groups. **Figure 7** shows the three most populated groups.

#### 5.4.3 Aggregated data: statistics and charts

**Figure 8:**
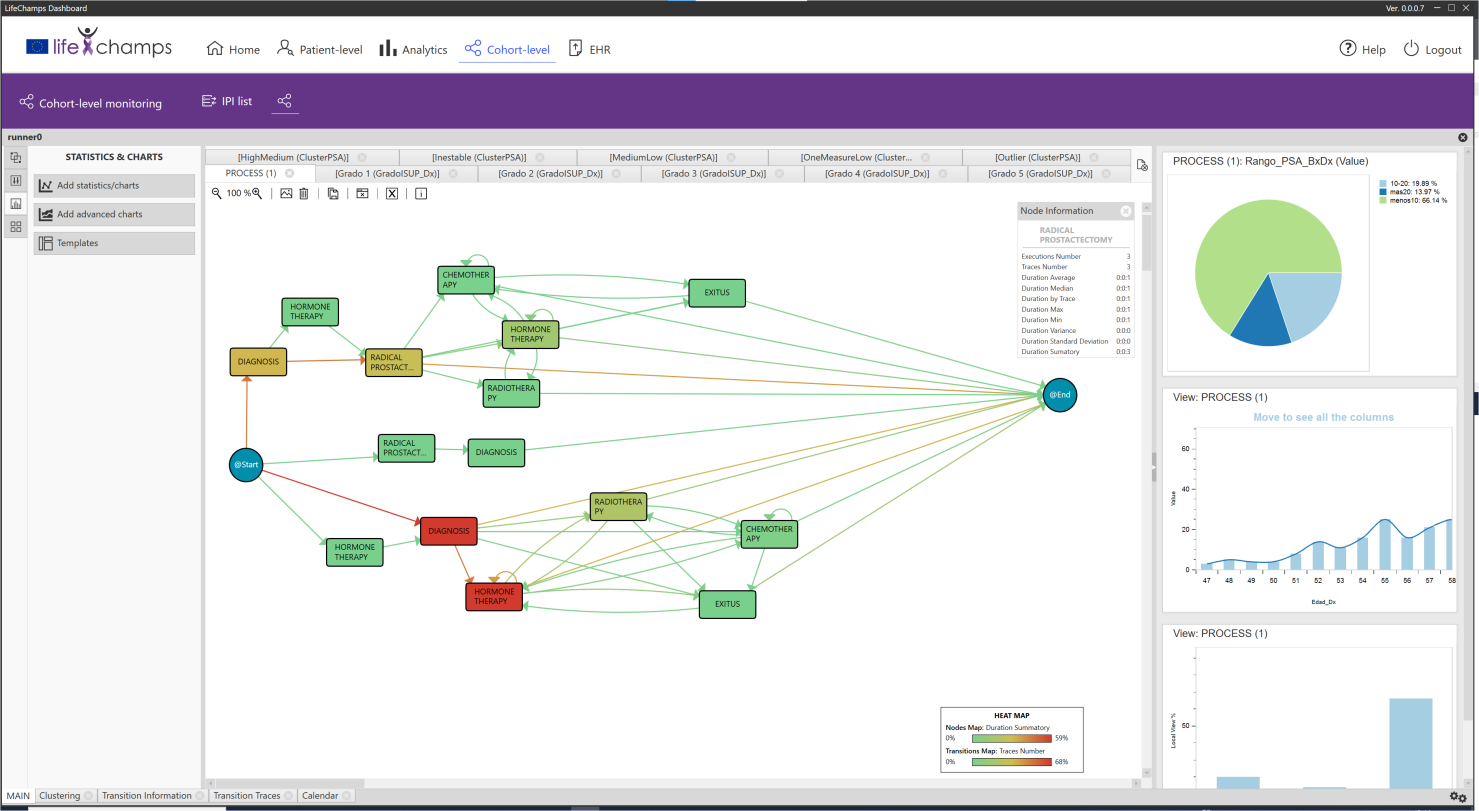
Statistics. IPI showing the statistics for Gleason Grade Group 1 about the PSA (ng/ml) range at diagnosis (less than 10, 10–20, more than 20), age at diagnosis, and treatment type for all patients.

The dashboard also incorporates statistical and chart features. Using patients’ aggregated data, it is possible to integrate statistics and charts into the IPI, such as graphs, tables, pies, or histograms. An example is included in **Figure 8**, where statistics about the percentage of patients at each PSA range at diagnosis are added.

### 5.5 Extended visualization

Process Mining Enhancement algorithms extend the information value of a process model using color gradients, shapes, or animations to highlight specific information in the workflow, providing an extra data visualization layer. With this in mind, the tool integrates features that allow users to add different layers to the existing process views, highlighting information that may be interesting for the analysis. The concrete features to be added to the IPI were analyzed and agreed upon with the HCPs during the Data-Rodeo sessions. The incorporated options are explained below.

#### 5.5.1 Enhancements

This functionality uses metadata from the process—nodes and arrows—to enhance it by adding different layers to the existing process. This tool improves the result’s understandability and professionals’ confidence in it, as the HCPs can play with the other options to discover the most appropriate IPI view. Depending on the option selected in the dropdown map, heat maps or difference maps are applied to the process views represented in the central perspective of the IPI. When *Heat* option is selected, the options to apply the heat maps are displayed. Then, the criteria with which the nodes and transitions are colored can be selected. **Table 4** presents the complete list.

**Table 4:** Enhancements for nodes and transitions.

Criteria	Description
Execution Number	The number of executions that passed through that node or transition.
Trace Number	The number of traces that have passed through that node or transition.
Duration summation	The sum of the time of all the executions that have passed through this node. The duration sum of all the executions associated with that transition, counting the time spent at the node where the transition was born.
Duration by Trace	For nodes, the time spent in a node. Within the same episode, the time a patient is in a concrete node is divided by the total trace time. For nodes, the time at the node of all runs associated with that transition, divided by the number of traces that pass through the transition.
Duration Average	Average duration of all executions that have passed through a node/transition.
Duration Median	The median duration of all executions that have passed through a node or associated with that transition.
Full Duration Average	The average duration of time at the node of all the executions associated with this transition, understanding the transition as the time between the beginning of node X and the beginning of node Y.
Full Duration Median	The median duration of time at the node of all the executions associated with that transition, understanding the transition as the time between the start of node X and the start of node T.

**Figure 9:**
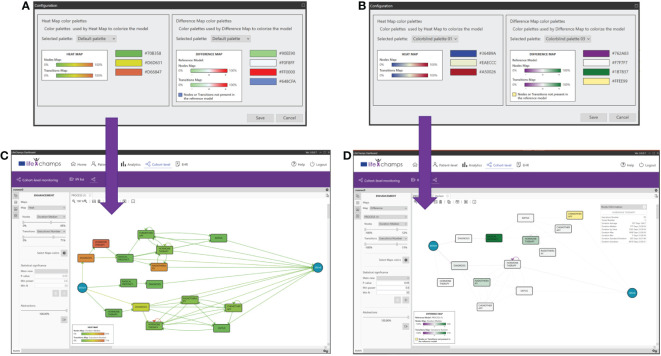
Enhancements. Color maps, palettes, and their application for enhancements. **(A)** Heat map colors, **(B)** heat map colors for colorblind, **(C)** enhanced model resulting from palette A, and **(D)** difference model resulting from palette B.

Color maps are applied to nodes and transitions based on the chosen criteria. Several color palettes are available in the dashboard to cover different HCPs’ needs, such as color palettes specially designed for users with color blindness (see **Figure 9**). In particular, heat map pallets use three colors for the gradient, and difference map color pallets use four colors, three for the gradient and one for the nodes or transitions not present in the reference model. Moreover, five of those nine color pallets are specially designed for users with color blindness. We can consider the following example to understand how the enhancement feature works. The Execution Number option is selected from the transitions criteria and the Duration Median for the nodes. With these options and considering the Execution Number, the maximum and minimum values of all the executions per transition are calculated. The correspondence with the colors is calculated based on the selected heat map color palette. For the heat map in **Figure 9C**, the greenest color in transition, as opposed to red, represents a larger number of cases in a transition. In contrast, the greenest paint in nodes means higher time in a median in the stage. When modifying the bars with the %, those nodes that have less than, for example, 20% of the total Execution Number are colored in green, and those nodes that have more than, for instance, 80% of the total are colored in red.

When *Differences* between maps is selected, the difference maps are calculated, taking the current process view and the one chosen against which the comparison is being performed as a reference and using the difference map color selected (see **Figure 9B**). For example, the fewer Execution Number difference between both process views, the greener it is shown (for this concrete difference color pallet map). On the other hand, the more Execution Number difference, the purple it is colored (for this concrete difference color pallet map). On the contrary, if the difference is almost zero, the node is shown whiter (for this concrete difference color pallet map; see **Figure 9D**).

#### 5.5.2 Node and transition information

**Figure 10:**
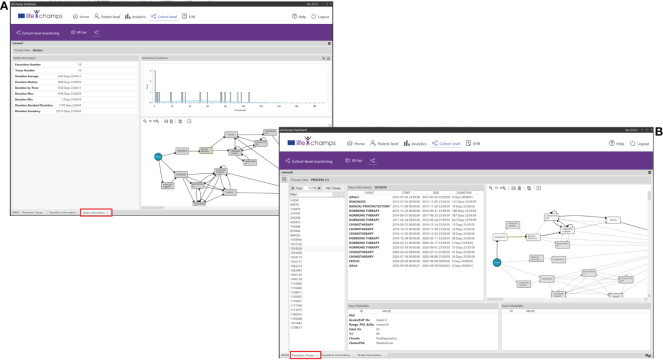
Perspectives. IPI perspective including the **(A)** Node and Transition Information, and the **(B)** Node and Transition Traces.

The *Node Information* and *Transition Information Perspectives* of the IPI present information about the process itself, in this case, about the selected node or transition of the working process view. They display statistics about traces, such as duration average, median, by patients, max., min., and variance, among others. See **Table 5** for the complete list of statistics. This information also presents a histogram with the event duration for the different patients with the Gaussian distribution, including the number of patients’ executions and the time taken for each, and the process view where the node/transition selection was made. Following the IPI, **Figure 10A** shows the *Radical Prostatectomy* node information with all its statistics.

**Table 5:** Statistical information associated to the processes.

Statistic	Meaning
Execution Number	Number of executions of the selected node/transition
Trace Number	Number of traces that traverse the selected node/transition
Duration Average	Average duration of all executions of the selected node/transition
Duration Median	Median duration of all executions of the selected node/transition
Duration by Trace	All-time execution of the concrete node/transition divided by the number of traces that traverse the selected node/transition
Duration Max	Maximum duration among all the executions of the selected node/transition
Duration Min	Minimum duration among all the execution of the selected node/transition
Duration Variance	Time execution duration variance of the selected node/transition
Duration Standard Deviation	Standard deviation of all time execution duration of the selected node/transition
Duration Summation	All execution time summation for the selected node/transition

### 5.6 From the process to the individuals

We have also incorporated the possibility of navigating from the process to the individuals into the dashboard. The *Nodes Traces Perspective* of the IPI displays the list of patients (traces) who traverse a selected node. Selecting a concrete patient in this view makes it possible to see the complete process and metadata. Similarly, the *Transition Traces Perspective* of the IPI displays the same information but considers the list of patients who traverse a selected transition. With these perspectives of the IPI, HCPs have complete knowledge of a concrete patient in a given context. **Figure 10B** shows patients who traverse the definite transition from the node *Diagnoses* to *Radical Prostatectomy*. The perspective includes the trace information for the selected trace, which means the whole process for the concrete patient, and the trace metadata, which means the aggregated data we have considered for the IPI, such as age or ISUP Grade Group at diagnosis.

## 6 Discussion

In this work, we proposed a novel analytical interactive Process Mining tool integrated into the LC Dashboard to monitor cancer patients’ progress. The tool uses Process Mining techniques to analyze cancer patients’ processes using data from different sources, such as clinical events and self-reported information. Process Mining techniques can potentially infer relevant information from data so that other processes can be discovered, compared, and highlighted. Following the Interactive Process Mining methodology through the application of a Data-Rodeo, we followed a co-creation process that involved experts from the Process Mining world and health experts in the cancer field. This process included the interaction between the two field experts through several sessions and the analysis of a data set for 1,267 unique patients with a prostate cancer diagnosis. As a result, we have developed a customized tool based on PMApp and a PI for comprehending prostate cancer patients’ evolution through their care process.

The tool presents results in the form of IPIs, as advanced views representing current processes, allowing their analysis by HCPs. The analytical Interactive Process Mining part of the LC Dashboard presents data at the cohort level with a novel focus, the care processes based on real-world data. Compared with previous works in clinical dashboards for clinical decision support, it supposes a step forward, complementing classical statistical analysis with the disease process perspective thanks to the IPIs. As explained in *Section 4*, several tools showed a proven capability to present data aggregated about individual patients and incorporate statistical analyses, but with poor or nonexistent AI technique integration to analyze data from a temporal perspective. The tool presented in this work includes Process Mining techniques to infer actual processes and present understandable and navigable results visually.

The tool, combined with the IPI, allows HCPs to perform their analysis, looking for different types of patients, considering the appropriate variable when pertinent, looking for different trajectories and behaviors, grouping patients according to them, etc. In this manner, we support them in inquiring about the most relevant clinical perceptual questions and the best views that help them in better processes’ perception and assessment. We also stated that few works had been done in the cancer field and specifically to monitor cancer patients’ progress. The analytical tool is fully adapted and customized to the problem. It has been co-designed and co-created with the target end-users, strengthening its value and use. The tool also allows the experts to navigate behind the model and discover the features and specificities of the process. Although cancer is very complex and its treatment depends on many variables, the tool and IPI offer new analytical and visualization possibilities, considering data from the process perspective. Although a complete evaluation of the tool regarding its usability and acceptance will be performed after the production phase that will take place in the context of the LifeChamps project with a 3-month duration, during the research phase, HCPs have become familiar with the tool, letting us collect preliminary insights into these concepts. HCPs found the analysis of the patient’s progress very valuable and innovative from a temporal perspective, as well as the possibility of co-creating both the tool and the IPI. However, they also mentioned some aspects to be improved. In particular, they stress the need for training using meaningful examples in the cancer field from the beginning, as the tool is not always intuitive. It is worth mentioning that HCPs gained confidence in the tool use during several Data-Rodeo sessions, improving their overall satisfaction with the tool and the IPI. Moreover, we incorporated a help section to facilitate its use and comprehension. However, this should be considered when comprising other HCPs in the study.

The results presented in this paper suppose the departure for a more ambitious scenario within the LifeChamps project. The project roadmap includes the validation of the LC Dashboard in pragmatic and clinical trials and conditions. This validation is being implemented through four feasibility pilots in four countries (Greece, Sweden, United Kingdom, and Spain), following up on 250 older cancer patients (aged 50 years old) diagnosed with prostate, breast, or melanoma cancer. During this project stage, the LC Dashboard will be installed in the site’s facilities and validated by HCPs of the four clinical sites participating in the study. During this process, more Data-Rodeo sessions will be performed to build a set of new IPIs with clinical relevance for all the pilot sites, considering their concrete characteristics and context. Accordingly, the Analytical Interactive Process Mining tool for monitoring cancer patients’ progress of the LC Dashboard will include the new IPIs and, if pertinent, new views or information based on the HCPs’ feedback during the different Data-Rodeo sessions. It is worth mentioning that the tool is ready to consume data from the EHR of the four organizations, not only the one participating in the current study. Moreover, it is also ready to consume data from other data sources included in the LifeChamps project. Although we have just incorporated data from an EHR in this work, the dashboard is ready to consume data from different sources during the pilots. These data include patients’ self-reported information through a mobile App, Patient Reported Outcome Measures (PROMs), and data from sensors and personal devices. This strategy will allow us to build novel and enriched IPIs to analyze cancer patients’ evolution from cohort and individual perspectives. During this process, the Data-Rodeo methodology will permit Process Mining experts to translate HCPs’ questions and hypotheses into novel approach views to be presented to HCPs. At that moment and coinciding with the production phase, the IPIs will be distributed through the LC Dashboard. Then, HCPs will use the dashboard in their daily practice to empower their decisions.

One of the tool’s strengths is that it has been co-created and co-designed with health experts. At the same time, it supposes one of its main challenges. The health experts’ involvement in all the procedures is paramount to ensure the clinical utility of the IPIs and the utilization of the dashboard. However, this involvement is not always easy or direct, and much effort should be placed into engaging them. Moreover, the tool and the implemented techniques have a high learning curve due to the paradigm change associated with the analysis. During the different phases, the preparation and the research, the communication between the two expert fields (process miners and HCPs) was not straightforward. However, the involvement of the same experts in all sessions was even more critical when talking about HCPs. It is worth mentioning that HCPs gained tool understanding through consecutive sessions. Consequently, they were able to determine the clinical needs, and process miners could translate them into the dashboard and IPI. In general, it was difficult to engage them because of their limited time. Many times, any new initiative is foreseen as an added workload for HCPs if they do not clearly see the benefits in their work and patients. Moreover, not all HCPs have the same degree of expertise when talking about new analytical techniques or have the same degree of interest. In our case, the involvement of the biomedical engineer in the process was crucial, as he acted as a bridge between the two worlds (healthcare and Process Mining), and had a good understanding of the hospital processes and systems, and the diseases. In this way, we managed to mitigate the limitation. When this possibility is not available, it is recommended to describe and agree on the approach collaboratively between HCPs and process miners from the beginning. The next phase, production, will give us more insights into the best practices to approach HCPs and acceptance.

The results demonstrated in this work present some limitations. The most important limitation is the remaining evaluation by a higher number of end-users. The tool has been presented to some HCPs, and only one has participated in the IPI development. This evaluation will be carried out in the context of the LifeChamps project for 3 months. After this evaluation, we will consider their inputs and their appropriateness to be incorporated into the tool. Moreover, during this production phase, we will collaborate with other HCPs from the breast and melanoma fields to design and develop new IPIs. However, due to the complexity of these diseases, they will suppose further case studies out of the scope of unique work.

Healthcare organizations’ systems are very heterogeneous and *ad hoc*. Thus, data ingestion always assumes a limitation for tools analyzing clinical data, and the tool presented in this paper is no exception. Considering this, we adapted our tool to be compatible with the four clinical settings participating in the LifeChamps project. However, this could not be enough to widen its application to other institutions. One of the potentialities of the interactive methodology is to consider the concrete data ingestion particularities during the initial sessions and adapt the tool accordingly, as PMApp supports this customization. PMApp could access the raw database directly by building query languages. If there is no access directly to the data, it is possible to transform the raw database into CSV (Comma-Separated Values) to facilitate its processing. This will always result in a customized tool, but we consider this as a strength rather than a weakness.

## Data availability statement

The data analyzed in this study is subject to the following licenses/restrictions: Data owner shared the data only in the context of the present study, no other analysis are allowed. Requests to access these datasets should be directed to zoevara@itaca.upv.es.

## Author contributions

ZV-R, CF-L and VT contributed to the conception and design of the work. GC organized the database. GC and BV contributed to the design and validation of the results. CF-L performed the development of the tool. ZV wrote the first draft of the manuscript. CF-L, GC, and AB wrote sections of the manuscript. All authors contributed to the manuscript, and revised, read, and approved the submitted version.
